# A Label-Free Fluorescence Aptasensor Based on G-Quadruplex/Thioflavin T Complex for the Detection of Trypsin

**DOI:** 10.3390/molecules27186093

**Published:** 2022-09-18

**Authors:** Pan Gu, Yangfan Lu, Shanni Li, Changbei Ma

**Affiliations:** School of Life Sciences, Central South University, Changsha 410013, China

**Keywords:** thioflavin T, trypsin, cytochrome c, DNA aptamer, label-free

## Abstract

A novel, label-free fluorescent assay has been developed for the detection of trypsin by using thioflavin T as a fluorescent probe. A specific DNA aptamer can be combined by adding cytochrome c. Trypsin hydrolyzes the cytochrome c into small peptide fragments, exposing the G-quadruplex part of DNA aptamer, which has a high affinity for thioflavin T, which then enhances the fluorescence intensity. In the absence of trypsin, the fluorescence intensity was inhibited as the combination of cytochrome c and the DNA aptamer impeded thioflavin T’s binding. Thus, the fluorescent biosensor showed a linear relationship from 0.2 to 60 μg/mL with a detection limit of 0.2 μg/mL. Furthermore, the proposed method was also successfully employed for determining trypsin in biological samples. This method is simple, rapid, cheap, and selective and possesses great potential for the detection of trypsin in bioanalytical and biological samples and medical diagnoses.

## 1. Introduction

Trypsin, one of the major and well-known proteolytic enzymes secreted by the pancreas, is integrally involved in many biological processes, such as protein digestion and the regulation of pancreatic function [1]. Trypsin is secreted from the acinar cells of the pancreas to the gastrointestinal tract via the pancreatic duct, where this enzyme is activated by enterokinase or autocatalysis; the resultant peptides are further digested by a variety of exopeptidases, and, subsequently, the small peptic fragments are absorbed in the intestine [2]. Abnormal trypsin levels are associated with different diseases, including pancreatitis (both chronic and acute) [3,4,5,6,7], cancer [8,9,10], inflammation [11,12,13], and septicemia [14]. The serum of a healthy individual contains 250 ± 100 ng/mL of trypsin, while patients with pancreatitis or pancreatic cancer may show a higher concentration of serum-trypsin, i.e., 1400 ± 600 ng/mL [15,16]. Patients with nutritional disorders typically exhibit serum-trypsin concentrations of less than 120 ng/mL [17,18]. For these reasons, trypsin has been successfully used as a biomarker for the diagnosis of pancreatic diseases and other health conditions [19,20]. Herein, a selective and sensitive detection method was developed that can accurately measure serum-trypsin levels in a wide concentration range and the presence of different interfering species.

Currently, various methods have been employed successfully for the detection of trypsin, including enzyme-linked immunosorbent assay (ELISA) [21,22], high-performance liquid chromatography (HPLC) [23], gelatin-based film technique [24], and colorimetric detection [25,26,27]. Nevertheless, the applicability of these traditional methods is compromised due to the time-consuming and complex sample preparation methods, high capital investment for instruments, significant recurring expenses for consumables, and the requirement of highly trained personnel. In particular, the ELISA method requires costly antibodies and involves expensive and complicated pretreatment methods. Environmental conditions, such as temperature and pH, greatly affect the stability of the antibodies and antigens. Thus, there is a need for the development of a simple, fast, and sensitive biosensor for the quantitative determination of trypsin. For the quantitative determination of trypsin, several biosensors have been developed based on different methods, such as Quartz Crystal Microbalance (QCM) [28] and fluorometric [29,30,31,32], photoelectrochemical, and electrochemical methods [33,34]. Among these methods, fluorometric methods are extremely popular owing to their inherent advantages, such as high sensitivity, simple instruments, rapid response, easy operation, and real-time detection. Until now, many fluorescence-turn-on-strategy-based assays have been developed for trypsin determination. Chen et al. reported a fluorescence turn-on method for the detection of trypsin with an inspiring detection limit of 0.004 μg/mL [35]. However, the linearity range of this method was narrow (from 0.01 to 2 μg/mL), which limited its application in clinical use. Huang et al. reported a trypsin detection method based on fluorescent probe HBI-GR; the probe was synthesized by combining the fluorophore (p-HBI) in green fluorescent protein (GFP) and guanine riboside (GR), and this method showed an excellent lower detection limit, i.e., 0.0282 ng/mL [31]. Wang et al. established a label-free and sensitive fluorometric procedure on the basis of fluorescence resonance energy transfer (FRET) between mercaptoundecanoic acid-functionalized gold nanoclusters (AuNCs) and gold nanoparticles (AuNPs) [29]. Wu et al. prepared protease-sensing, protein-conjugated QDs [36]. However, the applicability of these methods is compromised by several limitations, such as impractical clinical application, fluorescence labeling, high-cost materials, and the relatively high toxicity of quantum dots. Hence, a simple, sensitive, and practical quantitative method should be developed for determining trypsin.

The SELEX (Systematic Evolution of Ligands by Exponential Enrichment) method has been employed for the in vitro screening of the short segments of RNA or DNA single-stranded oligonucleotide sequences as aptamers [37,38]. They are equipped with an ability that frames unique three-dimensional structures via folding, including hairpin, pseudoknot, and G-quadruplex, to combine them with target molecules with a high affinity and high specificity [39,40,41]. The high affinity and specificity allow them to function as antibodies. Furthermore, aptamers are advantageous due to their low cost, high stability, and simple synthetic processes [42,43]. Therefore, aptamers are widely used for detecting different chemical compounds in protein research, drug analysis, virology, and food safety [44,45,46,47].

Hence, based on the high affinity between DNA aptamer and cytochrome c (cyt c) and the hydrolysis characteristics of trypsin [48], a simple, cost-effective, thioflavin T (ThT)-based, and quencher-free fluorescence method has been developed. Cyt c (a metalloprotein containing 104 amino acid residues) serves as a representative target for trypsin [49]. ThT-based fluorescent probes have been widely utilized in many fields due to their inherent advantages, such as sensitivity, convenience, and cost-effectiveness [50,51,52,53]. ThT can effectively bind to G-quadruplexes, resulting in an enhanced fluorescence signal [54,55]. The developed method has been successfully applied in determining trypsin in biological samples, and thus, this method has been shown to have promising applications in clinical use.

## 2. Results and Discussion

### 2.1. Principle of Trypsin Detection

**Figure 1:**
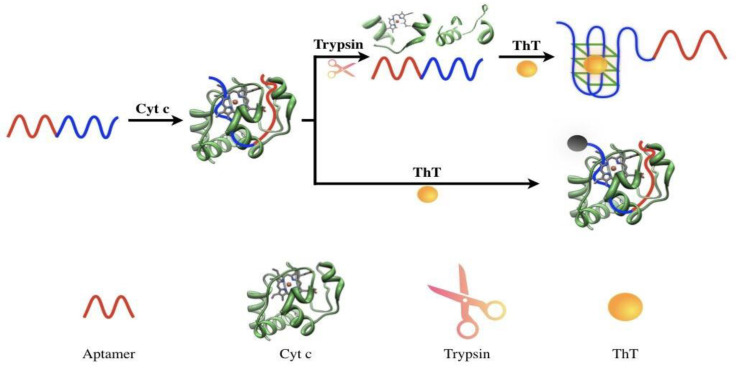
Schematic illustration of the fluorometric assay for detecting trypsin.

The principle of this assay is depicted in Figure 1. In this study, a DNA aptamer with high specificity to cyt c was selected through SELEX and then sequenced, synthesized, and characterized [48]. After the addition, the specific DNA aptamer quickly combined with cyt c (due to its high molecular weight). Subsequently, the steric hindrance prevented the linkage between ThT and the G-quadruplex part of the DNA aptamer, thus significantly reducing the fluorescence. In the detection system, trypsin can cleave cyt c into small peptide fragments, thus reducing the steric hindrance against the linkage and restoring the fluorescence. By measuring the degree of fluorescence recovery, the amount of trypsin can be determined.

### 2.2. Verification of the Feasibility of Trypsin Detection

**Figure 2:**
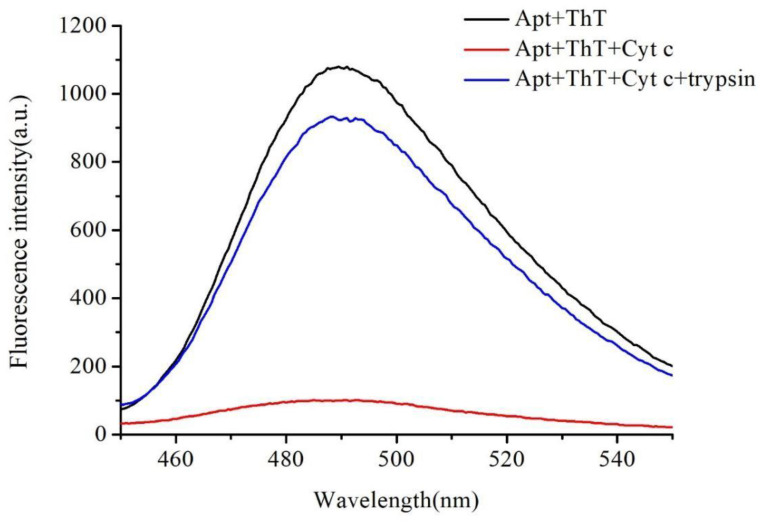
Feasibility of the proposed method: Fluorescence intensity of DNA aptamer-ThT; DNA aptamer-ThT with and without adding trypsin. All reactions were performed in 50 mmol/L Tris and 1 mmol/L KCl buffer at pH 7.5. In total, 50 μmol/L DNA aptamer, 1 mmol/L ThT, 10 mmol/L cyt c, 10 mg/mL trypsin, and 30 min of incubation time were employed. Fluorescence intensity was measured using an F-2700 with excitation at 490 nm, and the emission spectra were collected in the range of 450 to 550 nm.

The proposed method can detect strong levels of fluorescence at 490 nm of excitation from the G-quadruplex structure, which was formed by the association between the DNA aptamer and the ThT in buffer. However, no fluorescence was detected after the addition of cyt c, as the cyt c bonded to the DNA aptamer and prevented the formation of a G-quadruplex structure. The addition of trypsin restored the fluorescence. Trypsin cleaved the cyt c to small peptide fragments and assisted in the formation of a G-quadruplex structure (Figure 2). In summary, the results of this experiment demonstrated the feasibility of the proposed strategy for the determination of trypsin.

### 2.3. Optimization of Experimental Conditions

**Figure 3:**
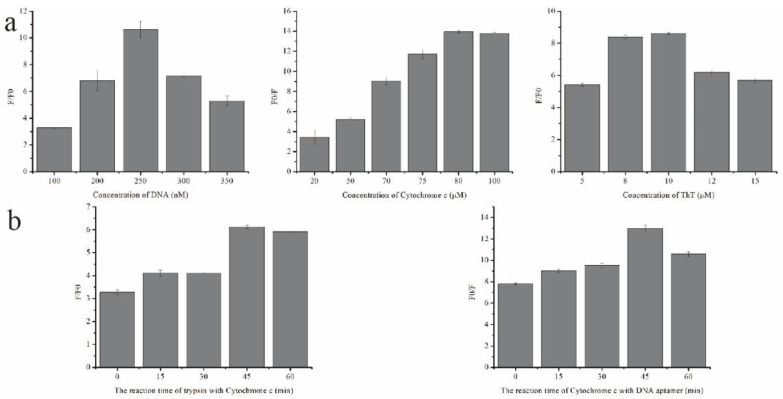
Optimizing the experimental conditions: (**a**) concentration of different experimental components, including DNA chain (1 mM ThT, 10 mM cyt c, and 10 mg/mL trypsin; 30 min of incubation time and a different concentration of DNA aptamer were employed), cytochrome c (250 nM DNA aptamer, 1 mM ThT, and 10 mg/mL trypsin; 30 min of incubation time and a different concentration of cyt c were employed), and ThT (250 nM DNA aptamer, 80 μM cyt c, and 10 mg/mL trypsin; 30 min of incubation time and a different concentration of ThT were employed); (**b**) reaction time for trypsin and cytochrome c (250 nM DNA aptamer and 80 μM cyt c with or without 10 mg/mL trypsin incubated within a different time range and then 10 μM ThT added for testing) and reaction time for the DNA chain (250 nM DNA aptamer with or without 80 μM cyt c incubated within a different time range and then 10 μM ThT added for testing).

Different parameters influence fluorescence intensity during trypsin detection. Not only the concentration of each component but also the reaction time are critical factors for a desirable fluorescence response [56,57]. At first, the concentration range of the DNA aptamer was optimized between 100 nM to 350 nM. As shown in Figure 3a, the optimal concentration of the DNA aptamer was 250 nM. We then applied the 250 nM DNA aptamer in the assay to optimize the concentration of cyt c from 20 μM to 100 μM (a), and the ratio of the increase in fluorescence intensity reached a plateau at 80 μM of cyt c (Figure 3a). Under 250 nM of DNA aptamer and the 80 μM cyt c condition, the concentration of ThT was optimized from 5 μM to 15 μM, and the optimal concentration was achieved at 10 μM (Figure 3a). Figure 3b shows that the fluorescent intensity increased with the increased time for the reaction between trypsin and cyt c. The fluorescent intensity reached a peak after 45 min when the binding between trypsin and cyt c reached equilibrium and a saturation point. Moreover, 45 min was also a suitable hybridization time for cyt c, the DNA aptamer, and trypsin. Thus, 45 min was chosen as the incubation time for the reactions of cyt c with the trypsin and DNA aptamer.

### 2.4. Quantitative Measurement of Trypsin

**Figure 4:**
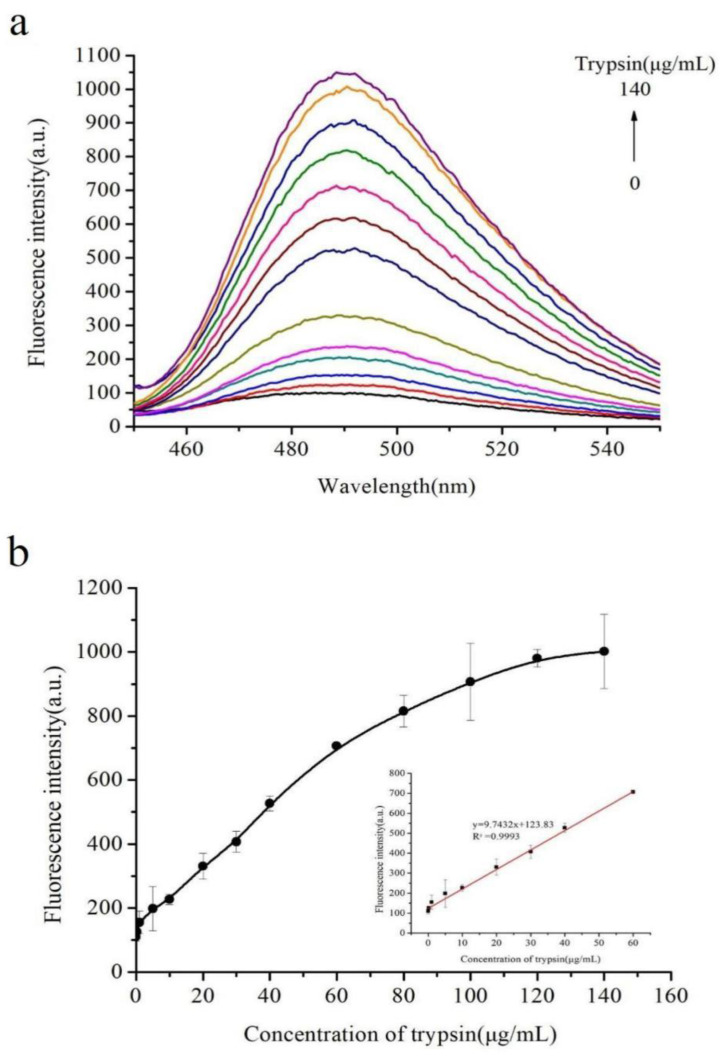
(**a**) Fluorescence emission spectra upon addition of trypsin at different concentrations, from 0 to 140 μg/mL. (**b**) Fluorescence signal in response to different concentrations of trypsin, showing the linear curve of the enhanced fluorescence intensity to the concentration of trypsin. The inset shows the linearity of the fluorescence intensity with respect to trypsin concentrations.

A series of concentrations of trypsin ranging from 0 to 140 μg/mL (0, 0.2, 1, 5, 10, 20, 30, 40, 60, 80, 100, 120, 140 μg/mL) was selected for evaluating the sensitivity of the proposed method. Figure 4a shows that the fluorescence intensity at 490 nm dynamically increased while increasing the concentration of trypsin. Figure 4b shows the relationship between the fluorescence intensity and the concentration of trypsin. Figure 4b shows that the fluorescence intensity possessed a linear relationship (R^2^ = 0.9993) with the concentration of trypsin in the range of 0.2–60 μg/mL, and the regression equation is Y = 9.7432X + 123.83, where Y is the fluorescence intensity at 490 nm and X is the trypsin concentration. The limit of detection of the proposed method was 0.2 μg/mL. Therefore, a simple, rapid, effective, and sensitive method has been established for determining trypsin. Furthermore, the proposed method showed a lower detection limit (0.2 μg/mL) and a wider linear range (0.2–60 μg/mL) than the previously reported trypsin detection methods (Table 1). In addition, the assay did not involve any synthetic or time-consuming procedures. Therefore, the proposed assay can easily and successfully quantify trypsin.

### 2.5. Study of Interferences

**Figure 5:**
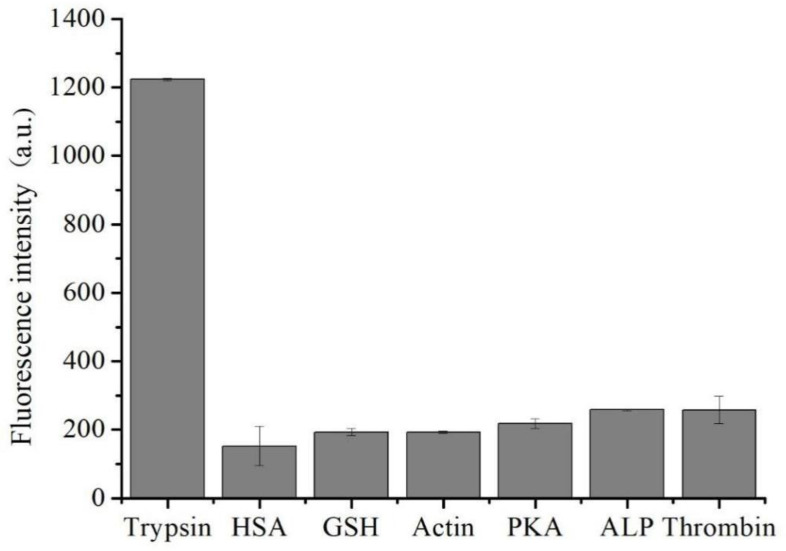
Selectivity of the assay. Selectivity of the proposed trypsin assay toward trypsin, HSA, GSH, Actin, PKA, ALP, and thrombin (60 μg/mL each).

Several proteins, such as HSA, GSH, Actin, PKA, ALP, and thrombin, were tested by the proposed assay under the optimized concentrations to investigate the selectivity of the method. Figure 5 implies that none of the proteins influenced the combination of DNA aptamer and cyt c. Therefore, the proposed assay is specific for the detection of trypsin, and the method possesses great potential in evaluating biological samples.

### 2.6. Trypsin Detection Assay in Real Samples

The method was applied to real samples, and diluted serum was added to the reaction system. When 15, 40, and 60 μg/mL of trypsin was added to the samples, the recovery rates were 103.9%, 91.8%, and 101.95%, respectively (Table 2). The result indicates that the trypsin detection method can be practically used in real samples.

## 3. Materials and Methods

### 3.1. Materials and Reagents

Trypsin was purchased from Sigma Aldrich (St. Louis, MO, USA). Standard solutions of trypsin were prepared daily by further diluting its stock solution (0.01 g trypsin was dissolved in 1 mL ultrapure water, and the concentration of trypsin was 10 mg/mL), which was stored at −20 °C. Human serum albumin (has), protein kinase (PKA), alkaline phosphatase, thrombin, glutathione (GSH), Actin, cyt c, and ThT were also purchased from Sigma Aldrich (St. Louis, MO, USA). The oligonucleotide cyto c apt (trypsin) was purchased from Sangon Biological Engineering Technology & Services (Shanghai, China). The DNA aptamer to cyt c was synthesized from Sangon Biotech Co. Ltd. (Shanghai, China), which had the following sequence: 5′-Fam-CCG TGT CTG GGG CCG ACC GGC GCA TTG GGT ACG TTG TTG CAA AAA GGG TTA GGG TTA GGG TTA GGG C-3′ [62]. The DNA sequence was dissolved in TE buffer and stored at −20 °C for further use. All solutions were prepared using ultrapure water, which was obtained through a Millipore Milli-Q water purification system (Billerica, MA), with an electric resistance >18.3 MΩ. All other chemicals were of analytical grade and purchased from Sinopharm Chemical Reagent Co. Ltd. (Shanghai, China).

### 3.2. Apparatus

All fluorescence measurements were performed on an F-2700 spectrophotometer (Hitachi, Tokyo, Japan) with excitation at 490 nm and emission at 450–550 nm for the cyt c apt. The excitation slits and emission slits were, respectively, set at 10.0 and 10.0 nm. Each experiment was carried out in a final volume of 100 µL.

### 3.3. Trypsin Detection Assays

In this study, 10 mM of cytochrome c and different concentrations of trypsin were mixed together in the reaction buffer (50 mM Tris, 1 mM KCl, and pH 7.5) and incubated at 37 °C for 45 min. Then, 250 nM of cyt c apt was added to the reaction solution and incubated at 37 °C for 45 min. Finally, 10 μM of ThT was added to the solution and kept at room temperature for 10 min. Fluorescence intensity was measured using an F-2700 with excitation at 490 nm, and the emission spectra were collected at the range of 450 to 550 nm.

## 4. Conclusions

In summary, a successful fluorometric method has been developed based on DNA aptamer, cyt c, and ThT for detecting trypsin. The proposed method exhibited high sensitivity toward trypsin, with a detection limit of 0.2 μg/mL under optimized conditions. Furthermore, this method is simple and cost-effective, without any labels or complicated operations. The proposed strategy was also successfully applied in detecting trypsin in serum samples, and satisfactory results were obtained. Therefore, the present strategy based on DNA aptamer and cyt c may be applied for detecting trypsin in bioanalytical and biological studies and medical diagnoses.

## Figures and Tables

**Table 1:** Comparison of different methods of trypsin determination.

Method	Material	LOD ^1^ (μg/mL)	Linear Range (μg/mL)	Reference
Colorimetric	Copper ion chemosensor	1.0	1.0–5.0	[25]
Colorimetric	Gold nanoclusters	0.6	0.9–1000	[26]
Fluorescent	Ag nanoclusters	0.06	0.7–4.0	[58]
Fluorescent	Gold nanoclusters	0.08	0.2–100	[59]
Fluorescent	Conjugated polyelectrolyte	0.2	0–2.5	[60]
Fluorescent	Graphene quantum dots	0.7	0–6.0	[61]
Fluorescent	DNA aptamer	0.2	0.2–60	This work

^1^ Note: LOD, limit of detection.

**Table 2:** Recovery experiments for the determination of trypsin in human serum samples.

Sample	Added (μg/mL)	Found (μg/mL)	Recovery (%)	R.S.D (%, *n* = 5)
1	15	15.59	103.9	5.8
2	40	36.72	91.8	4.1
3	60	61.17	101.9	2.9

## Data Availability

Not applicable.

## Sample Availability

Samples of the compounds are available from the authors.
